# Clinical and functional evidence for the pathogenicity of the *LRRK2* p.Arg1067Gln variant

**DOI:** 10.1038/s41531-025-00884-6

**Published:** 2025-02-23

**Authors:** Shen-Yang Lim, Tzi Shin Toh, Jia Wei Hor, Jia Lun Lim, Lei Cheng Lit, Azlina Ahmad-Annuar, Yi Wen Tay, Jia Nee Foo, Ebonne Yulin Ng, Kalai Arasu Muthusamy, Norlinah Mohamed Ibrahim, Khairul Azmi Ibrahim, Louis Chew Seng Tan, Jannah Zulkefli, Anis Nadhirah Khairul Anuar, Kirsten Black, Pawel Lis, Fei Xie, Zhidong Cen, Kai Shi Lim, Katja Lohmann, Shalini Padmanabhan, Dario R. Alessi, Wei Luo, Eng King Tan, Esther Sammler, Ai Huey Tan

**Affiliations:** 1 Division of Neurology, Department of Medicine, Faculty of Medicine, University of Malaya, Kuala Lumpur, Malaysia; 2 The Mah Pooi Soo & Tan Chin Nam Centre for Parkinson’s & Related Disorders, Faculty of Medicine, University of Malaya, Kuala Lumpur, Malaysia; 3 Department of Biomedical Science, Faculty of Medicine, University of Malaya, Kuala Lumpur, Malaysia; 4 Department of Physiology, Faculty of Medicine, University of Malaya, Kuala Lumpur, Malaysia; 5 Lee Kong Chian School of Medicine, Nanyang Technological University Singapore, Singapore, Singapore; 6 Laboratory of Neurogenetics, Genome Institute of Singapore, A*STAR, Singapore, Singapore; 7 Department of Neurology, National Neuroscience Institute, Singapore, Singapore; 8 Division of Neurosurgery, Faculty of Medicine, University of Malaya, Kuala Lumpur, Malaysia; 9 Department of Medicine, Faculty of Medicine, Universiti Kebangsaan Malaysia, Kuala Lumpur, Malaysia; 10 Department of Medicine, Hospital Sultanah Nur Zahirah, Kuala Terengganu, Terengganu Malaysia; 11 Immunogenetic Unit, Allergy and Immunology Research Centre, Institute for Medical Research, National Institutes of Health Complex, Ministry of Health Malaysia, Setia Alam, Malaysia; 12 Medical Research Council Protein Phosphorylation and Ubiquitylation Unit, University of Dundee, Dundee, UK; 13 Department of Neurology, Sir Run Run Shaw Hospital, Zhejiang University School of Medicine, Hangzhou, Zhejiang PR China; 14 Department of Neurology, The Second Affiliated Hospital, Zhejiang University School of Medicine, Hangzhou, Zhejiang PR China; 15 Institute of Neurogenetics, University of Luebeck, Luebeck, Germany; 16 The Michael J. Fox Foundation for Parkinson’s Research, New York, NY USA; 17 Division of Neuroscience, School of Medicine, University of Dundee, Dundee, UK

**Keywords:** Parkinson's disease, Parkinson's disease

## Abstract

*LRRK2*-related Parkinson’s disease (*LRRK2*-PD) is the most frequent form of monogenic PD worldwide, with important therapeutic opportunities, exemplified by the advancement in LRRK2 kinase inhibition studies/trials. However, many *LRRK2* variants, especially those found in underrepresented populations, remain classified as variants of uncertain significance (VUS). Leveraging on Malaysian, Singaporean, and mainland Chinese PD datasets (*n* = 4901), we describe 12 Chinese-ancestry patients harboring the *LRRK2* p.Arg1067Gln variant, more than doubling the number of previously reported cases (total *n* = 23, 87% East Asian, mean age of onset: 53.9 years). We determine that this variant is enriched in East Asian PD patients compared to population controls (OR = 8.0, 95% CI: 3.0–20.9), and provide supportive data for its co-segregation with PD, albeit with incomplete penetrance. Utilizing established experimental workflows, this variant showed increased LRRK2 kinase activity, by ~2-fold compared to wildtype and higher than the p.Gly2019Ser variant. Taken together, p.Arg1067Gln should be reclassified from a VUS to pathogenic for causing *LRRK2*-PD.

## Introduction

Monogenic forms of Parkinson’s disease (PD) include *SNCA*, *LRRK2*, *VPS35*, *RAB32*, and *CHCHD2*, which are associated with autosomal dominant PD, and *PRKN*, *PINK1*, and *DJ-1*, which are associated with autosomal recessive PD^1–3^. Of these, the most important in terms of global frequency is *LRRK2* causing *LRRK2*-PD^4^. All pathogenic variants in *LRRK2* result in hyperactivation of the LRRK2 kinase, conferring strategic therapeutic opportunities, where inhibition of this kinase has now taken center stage in genetically-informed clinical trials in PD^5^.

Currently, just over 20 variants in *LRRK2* are considered to be pathogenic or likely pathogenic for PD^6^. Some of these variants, such as p.Gly2019Ser and p.Arg1441Gly, are known to be enriched in specific global populations (African-Berbers and Ashkenazi Jews; and Spanish Basques, respectively)^3,4^, and others such as p.Asn1437Asp have been tentatively reported to be more common among Chinese but await replication^7^. The Chinese population is also believed to have a founder for a variant at the p.Arg1441 hotspot, where the arginine is substituted by a cysteine, the p.Arg1441Cys *LRRK2* variant, with several patients/families described from Singapore, Malaysia, and China^8–10^. Generally, however, monogenic forms of *LRRK2*-PD are believed to be rare in Asian populations with, for example, the overall most commonly reported *LRRK2* variant, p.Gly2019Ser, being almost completely absent^11^. In contrast, the “Asian” *LRRK2* risk variants are prevalent with p.Gly2385Arg and p.Arg1628Pro each being detected in ~5-10% of PD patients (vs. ~½ those frequencies in controls) in several Asian populations^11^.

A large number of *LRRK2* variants, numbering almost 200, are presently classified as variants of uncertain significance (VUS) (see https://www.mdsgene.org)^12^. Although in some cases co-occurrence with PD has been reported, data have been lacking from extended pedigrees (to assess co-segregation of the variant with disease), large case-control samples, and/or functional assays in model systems, to enable a more definitive determination of the pathogenicity of these VUS. In most settings, the latter are unavailable or expensive and complex to perform. Expectedly, VUS are more common in populations underrepresented in genetics research, and this further exacerbates global inequities in healthcare^3,13,14^.

Here, we aimed to decipher the pathogenicity of the *LRRK2* p.Arg1067Gln (p.R1067Q) variant (NM_198578.4, rs111341148, c.3200G>A), which authors have usually classified as a VUS^15–22^, by leveraging on large datasets of Malaysian, Singaporean, and mainland Chinese PD patients, and using a recently established analytic workflow to determine kinase activity of individual *LRRK2* variants in vitro and in vivo^6,23^. We further studied the clinico-demographic features of patients with this variant in these cohorts, and in the published literature.

## Results

### Identification of a Malaysian PD proband with the *LRRK2* p.Arg1067Gln variant

This work was initiated by the discovery of the p.Arg1067Gln variant in a Malaysian patient (PD-3402) of Chinese ancestry with early-onset PD (symptom onset aged 45 years). He was initially seen by a movement disorder neurologist (N.M.I.) in his early 50 s and found to have impaired upgaze, which was considered atypical for PD. The patient, however, had disabling motor fluctuations (with akinesia and stiffness during OFF, becoming wheelchair-dependent) as well as troublesome dyskinesias, taking a high dosage of PD medications, including levodopa 200 mg 2-hourly day and night. This led to a referral for consideration of deep brain stimulation (DBS) and reassessment at the University of Malaya (S.Y.L. and K.A.M.). Upgaze restriction during OFF periods was noted (improving when ON), but otherwise his condition was typical for PD, and neuroimaging did not reveal any significant abnormalities. Bilateral subthalamic nucleus DBS was performed successfully at the age of 55 years, resulting in resolution of the motor complications (fluctuations and dyskinesias) and marked reduction in PD medication requirement (now taking only levodopa 50 mg four times daily). The upgaze restriction was no longer observed post-DBS.

There was no history of PD in the immediate or extended family. Both elderly parents were apparently healthy and non-consanguineous, and he was the third among seven siblings (however, family members were not available for clinical assessment). Because of his young age at PD onset, clinical genetic testing via a gene panel analyzing 66 genes, including sequencing of the *LRRK2* gene (Hereditary PD and Parkinsonism Panel - see Supplementary Fig. 1) and multiplex ligation-dependent probe amplification (MLPA) was performed^24^. This did not reveal any known pathogenic or likely pathogenic variant or relevant copy number variations in the PD-related genes tested. However, what was found was the presence of a heterozygous p.Arg1067Gln *LRRK2* variant, which was interpreted as a VUS (PM2 and PP3 according to the criteria of the American College of Medical Genetics [ACMG])^23^, since the variant is rare in the general population (PM2, with only 30 heterozygous carriers reported among 806,813 individuals in gnomAD [v4.1.0]) and in silico predicted to be pathogenic with a CADD score^25^ of 31 (PP3, v1.7, https://cadd.gs.washington.edu). No other pathogenic/likely pathogenic variants or VUS were detected in *LRRK2*.

### Identification of additional p.Arg1067Gln variant carriers with PD and risk ascertainment in East Asian case-control samples

Building on the discovery in our Malaysian proband, we first conducted a systematic literature review and identified a total of 11 patients (8 East Asian consisting of 5 Chinese, 2 Japanese, 1 Korean, as well as 2 related Turkish patients and 1 Italian) with PD who had previously been reported to harbor the p.Arg1067Gln variant, which authors have usually classified as a VUS (Table 1)^15–22^.

**Table 1:** Clinico-demographic features of patients harboring the *LRRK2* p.Arg1067Gln variant

Paper/Cohort characteristics	Skipper et al. *Neurol*. 2005 (*n* = 1 from sample of *n* = 630 predominantly [88%] Chinese PD patients; absent in *n* = 630 matched controls)	Zabetian et al. *Mov Disord*. 2009 (*n* = 2 unrelated patients, from sample of *n* = 631 Japanese PD patients) (also *n* = 1 unaffected carrier aged 83, from sample of *n* = 1641 Japanese/Japanese-American controls)	Kessler et al. *Parkinsonism Rel Disord*. 2017 (*n* = 1 index patient plus her sister^a^, from sample of *n* = 91 predominantly Turkish patients)	Youn et al. *Neurobiol Aging*. 2019 (*n* = 1 patient, from sample of *n* = 70 unrelated Korean EOPD patients)	Yang et al. *Neurobiol Aging*. 2019 (*n* = 2 patients, from sample of *n* = 1456 sporadic Mainland Chinese PD patients; absent in *n* = 1568 matched controls)	Li et al. *Neurobiol Aging*. 2020 (*n* = 1 patient, from sample of *n* = 240 Mainland Chinese EOPD patients)	Zheng et al. *Mol Genet Genomic Med*. 2020 (*n* = 1 patient, from sample of *n* = 191 Mainland Chinese sporadic PD patients)	Salemi et al. *biomedicines*. 2023. (*n* = 1 patient, from sample of *n* = 126 Sicilian ancestry PD patients)	This report: Patients 1-3 (*n* = 3 patients, from sample of *n* = 1871 multiethnic Malaysian PD patients, of whom 1251 were Chinese) (PD-3402, PD-1785, & PD-330, respectively)	This report: Patients 4-7 (*n* = 4 patients, from sample of *n* = 2430 multi-ethnic Singaporean PD patients, of whom 2230 were Chinese) (P1785, P199, P694, & PD/LT 16, respectively)	This report: Patients 8-12 (*n* = 5 patients, across 3 families, from sample of *n* = 600 mainland Chinese PD probands; of whom 64 were from families with autosomal dominant PD or had ≥1 other affected relative with PD within 3 generations	Overall, *n* = 23 cases
Gender	M	F F	F F	F	M M	F	UNK	M	M M M	M F M M	F^b^ M^b^ M^c^ M^c^ F	13 M,9 F,1UNK
Age at motor onset/*Disease duration (yrs)*	48/*8*	46/*UNK* 59/*UNK*	31/*14* 36/*10*	48/*UNK*	53/*UNK* 45/*UNK*	39/*1*	UNK/*UNK*	78/*7*	45/*11* 62/*5* 64/*15*	61/*1* 62/*2* 71/*2* 74/*9*	40/*17* 78/*10* 55/*7* 60/*10* 31/*3*	Mean 53.9 (range 31-78)/*Mean 7.8 (1-17)*
Ancestry/Location of patients	Chinese/Singapore	Japanese/Japan	Turkish/Turkey	Korean/South Korea	Chinese/South Central China	Chinese/West China	Chinese/East China	Sicilian (Caucasian)	Chinese/Malaysia	Chinese/Singapore	Chinese/China	17 Chinese, 2 Japanese, 1 Korean, 2 Turkish, 1 Sicilian (Caucasian)
Family history (of proband)	✗	✗ ✗	✓^a^	✗	✗ ✗	✗	UNK	UNK	✗ ✗ ✗	✗ ✗ ✗ ✗	✓ (paternal uncle) ✓ (1 sister and 2 brothers) ✓ (father)	4✓,14✗,2UNK
Overall comments/Other notes	The patient was said to be “similar to typical PD”^d^	The patient had clinically definite PD^e^ and did not report any NMS	The index patient did not report any NMS	Cohort patients were Dx using^f^	Cohort patients were Dx using^d^ or^f^	Cohort patients were Dx using^d^	Cohort patients were Dx using^f^	Cohort patients were Dx using^f^	Cohort patients were Dx using^d^. Patients 1, 2 and 3 were tested by Invitae gene panel, NBA, and CENTOGENE gene panel, respectively	Cohort patients were Dx using^d^	Cohort patients were Dx using^d^ or^f^	

✓ Present, ✗ Absent, *Dx* Diagnosed,  *EOPD* Early-onset Parkinson's disease, *F* Female, *M* Male, *NBA* NeuroBooster Array genotyping platform, *NMS* Non-motor symptoms, *PD* Parkinson’s disease, *UNK* Unknown.

**Fig. 1:**
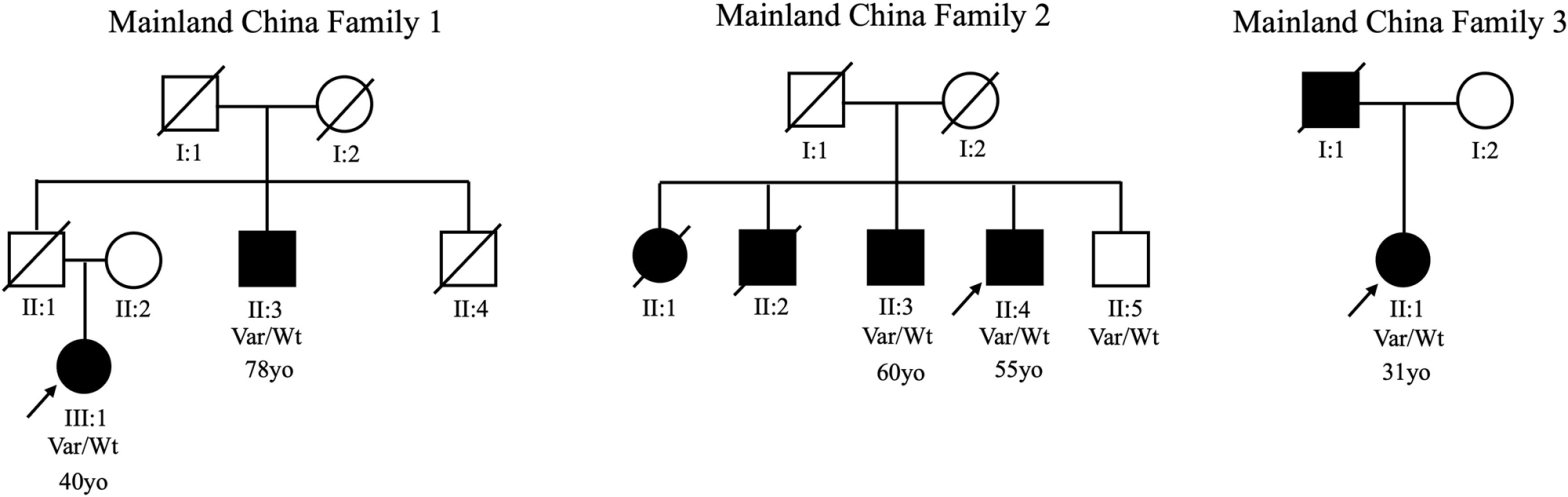
Family pedigrees of the three probands from mainland China with a positive family history of Parkinson’s disease. There is partial evidence for co-segregation (with the p.Arg1067Gln variant detected in five individuals affected with PD), however with seemingly incomplete penetrance (the youngest sibling in Family 2 [II:5] being clinically unaffected when assessed at age 63 years). The age of onset is written below each affected individual, where available.

^a^The proband’s father was also reported to have suggestive symptoms; testing of 4 other clinically unaffected sisters and other cousins appeared to show co-segregation of the variant with PD. Affected individuals (with the proband listed 1st) from Mainland China Families 1^b^ and 2^c^ (Fig. 1 depicts the family pedigrees). Using the United Kingdom (UK) Brain Bank^d^, Calne^e^, or International Parkinson and Movement Disorder Society (MDS)^f^ clinical diagnostic criteria for PD.

Noting the high prevalence of this variant among East Asians in previous reports, we interrogated our combined Malaysian, Singaporean, and mainland Chinese PD datasets (total *n* = 4901, of whom 4081 were of Chinese ancestry), and identified nine additional p.Arg1067Gln variant-positive PD probands of Chinese ancestry, apart from our Malaysian Chinese proband (frequency=0.0025; *n* = 10/4081) and two additional clinically affected family members. Clinico-demographic data of all 23 previously reported and newly identified p.Arg1067Gln variant carriers with PD are summarized in Table 1, while more detailed motor and non-motor features are summarized in Supplementary Table 1. Mean age of onset of these variant carriers was 53.9 ± 14.3 years (range:31 to 78 years); 4 out of 18 probands reported a positive family history (22.2%). The family pedigrees of the three probands from mainland China with a positive family history of PD are summarized in Fig. 1, providing partial evidence for co-segregation, albeit with seemingly incomplete penetrance.

In the gnomAD database (v4.1.0), the allele frequency of the p.Arg1067Gln variant in East and South Asians is 0.000156 and 0.000121 respectively. The variant is comparatively rare in other populations (ranging from <0.00001 in Europeans to 0.0000338 in Ashkenazi Jews), with an aggregated population allele frequency of 0.00001859. In the All of Us database (https://researchallofus.org/), the overall allele frequency is 0.000014, with the highest frequency seen again in East Asians (0.000372), compared to 0.00008 in Europeans. There are no homozygotes reported in any of the genomic databases. Comparison of mutant allele frequencies in our Chinese-ancestry PD patients vs. gnomAD East Asian controls plus Malaysian Chinese controls (*n* = 22,427 and 307, respectively, total *n* = 22,734) yielded an odds ratio for PD of 8.0 (95%CI: 3.0–20.9, *P* < 0.0001) (Supplementary Table 2), fulfilling the ACMG PS4 criterion for “strong” evidence of pathogenicity.

Haplotype analysis in the seven Malaysian and Singaporean carriers, 307 Malaysian East Asian (EAS) controls, and 1220 Malaysian EAS PD patients negative for p.Arg1067Q, was performed with forty-one single nucleotide polymorphisms (SNPs) selected from Neurobooster Array (NBA)^26^, CENTOGENE, and whole exome datasets over a 140kbp interval across the p.Arg1067Gln variant. The p.Arg1067Gln variant did not appear to be located on any shared disease haplotype among the seven carriers, or on any rare sub-haplotype based on 18 of the 41 SNPs with an MAF of 0.01 (Supplementary Table 3), and the haplotypes of the seven carriers were similar to the haplotypes seen in the controls or p.Arg1067Gln-negative patients (Supplementary Table 4). With whole genome data from two of the three probands from mainland China, finer mapping around the p.Arg1067Gln variant with additional SNPs indicated that there appeared to be an approximate 36.6kbp shared haplotype between the two probands, encompassed by SNPs rs139952019 (chr12:40276902, G>A) and rs11564178 (chr12:40313477G>A), which was not present in ten PD patients from mainland China negative for the p.Arg1067Gln variant (Supplementary Table 5).

### Functional assessment of the *LRRK2* p.Arg1067Gln variant

As previously shown, LRRK2 kinase pathway activity can be assessed by measuring phosphorylation levels of LRRK2 substrates e.g., Rab10 at threonine 73 either in vivo in human neutrophils or monocytes isolated from fresh peripheral blood, or in a robust cellular overexpression system^6,27,28^. In the cellular overexpression system, LRRK2 kinase hyperactivation is defined as pRab10Thr73 elevation of 1.5-fold compared to LRRK2 wildtype as before and this is about the level of activation seen with the common *LRRK2* p.G2019S variant^6^.

**Fig. 2:**
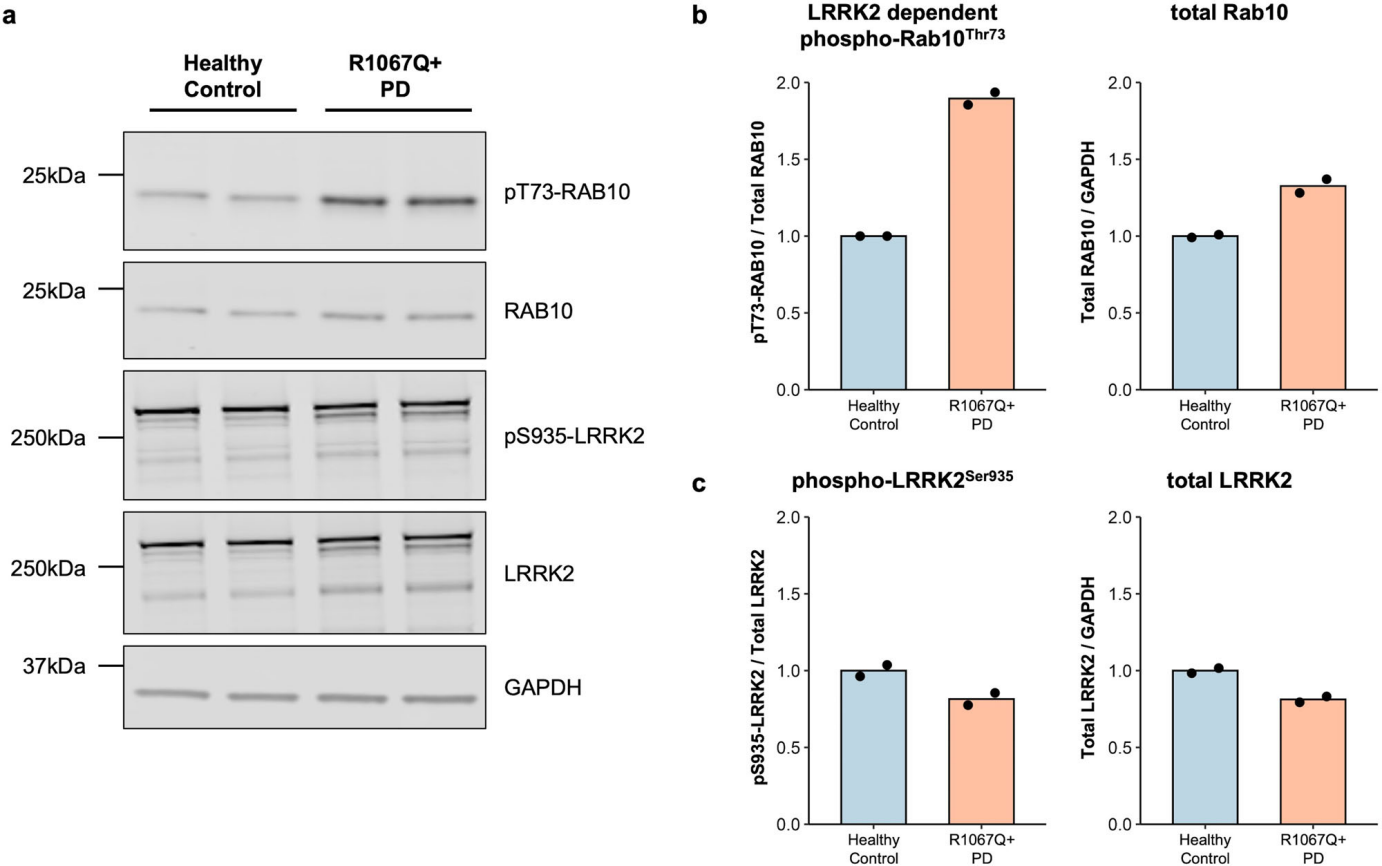
LRRK2 kinase hyperactivity in vivo due to the presence of the *LRRK2* p.Arg1067Gln (p.R1067Q) variant. Monocyte lysates were analyzed by quantitative immunoblotting (**a**). Quantified immunoblotting data are presented as ratios of phospho-Rab10^Thr73^/total Rab10 and total Rab10/GAPDH (**b**), and phospho-LRRK2^Ser935^/total LRRK2 and total LRRK2/GAPDH (**c**), normalized to the average values obtained from the healthy control. The experiments were performed in duplicates, with each data point representing a technical replicate. LRRK2-dependent Rab10 phosphorylation (phospho-Rab10^Thr73^) as a readout for LRRK2 kinase activity was increased in the monocytes derived from the patient carrying the p.Arg1067Gln variant compared to the control.

**Fig. 3:**
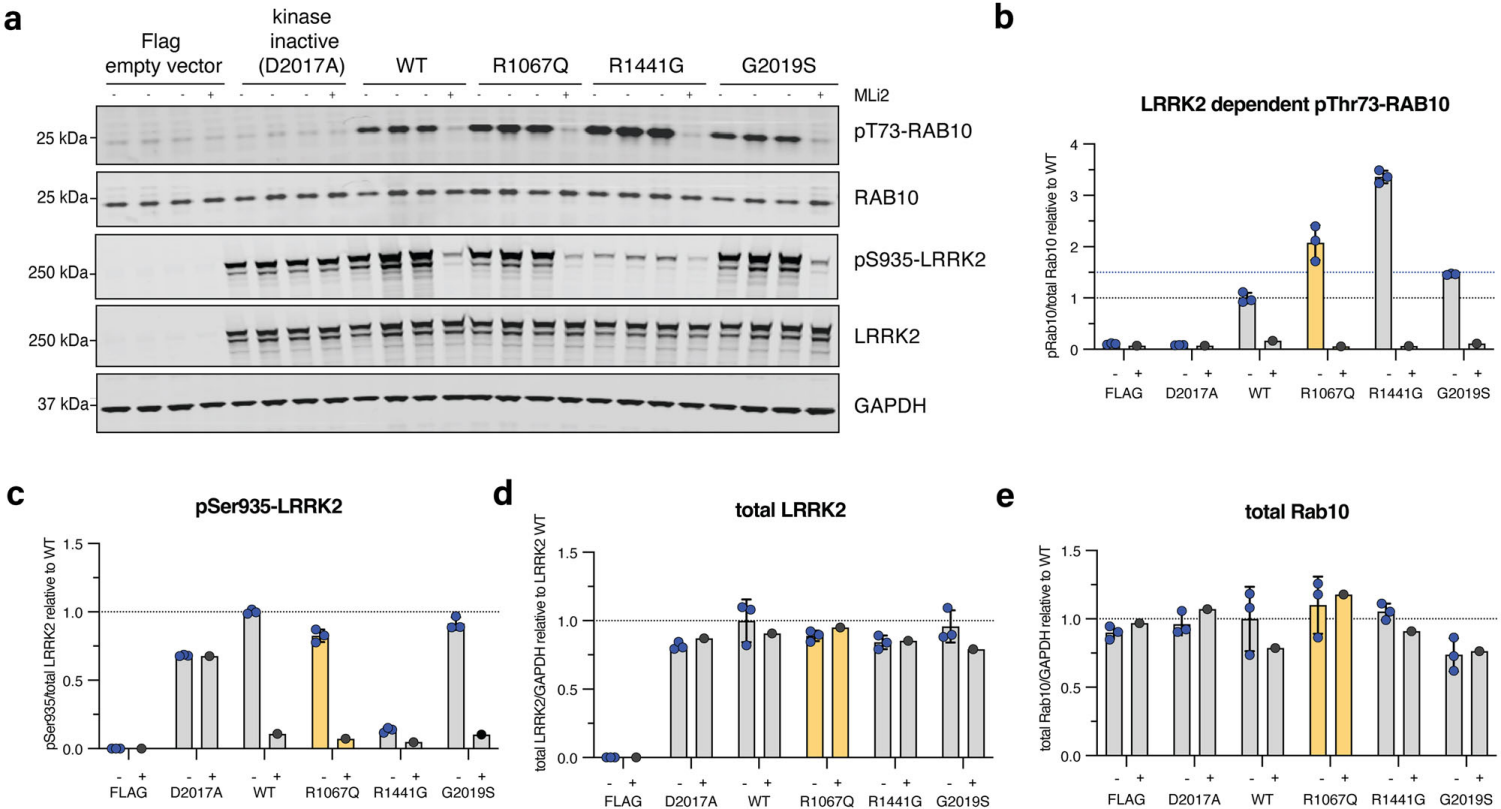
LRRK2 kinase activity of the *LRRK2* p.Arg1067Gln variant compared to p.Gly2019Ser and p.Arg1441Gly in a cellular overexpression system. In vitro characterization of the *LRRK2* p.Arg1067Gln variant in comparison with the common *LRRK2* p.Gly2019Ser and p.Arg1441Gly variants in an established HEK293 overexpression system, followed by LI-COR Odyssey immunoblotting and quantification of LRRK2 kinase activity relative to LRRK2 wildtype (wt) **(a)**. Three independent biological replicate transfection experiments were performed including one where cells were treated with and without the specific LRRK2 kinase inhibitor MLi-2 (200 nM for 1.5 h). Each (-) lane represents a biological replicate. LRRK2-dependent phosphorylation of endogenous Rab10 at threonine 73 (pRab10^Thr73^) was used as a readout for LRRK2 kinase activity, and the LRRK2-specific small molecule inhibitor MLi-2 to demonstrate LRRK2 kinase dependency of pRab10^Thr73^ as before. LRRK2 kinase hyperactivation was defined as pRab10^Thr73^ elevation of 1.5-fold compared to LRRK2 wt as before (blue dotted line) (**b**). Each datapoint represents a biological replicate experiment. p.Arg1067Gln showed LRRK2 activation of 2.4-fold, p.Gly2019Ser of 1.5-fold, and p.Arg1441Gly of 3.4-fold compared to LRRK2 wildtype (**b**). Expression levels of biomarker phosphorylation of LRRK2 Ser935 (**c**) and overexpressed LRRK2 (**d**) as well as endogenous levels of Rab10 (**e**).

We found a ∼2-fold increase in Rab10 phosphorylation in peripheral blood monocytes from patient PD-3402 in comparison to a healthy volunteer whereas total LRRK2, LRRK2 phosphorylation at Serine 935, and total Rab10 levels were relatively equal. This provides in vivo evidence for hyperactivation of the LRRK2 kinase in the presence of the p.Arg1067Gln variant in patient-derived cells (Fig. 2, Supplementary Fig. 2), and is in line with what we previously reported in our robust cellular overexpression system^6^. In the HEK293 overexpression assay^6^, the *LRRK2* p.Arg1067Gln variant also resulted in a ~2-fold higher LRRK2 dependent Rab10 phosphorylation level compared to wildtype LRRK2 (Fig. 3a). In parallel experiments, the common p.Gly2019Ser and p.Arg144Gly variants increased Rab10 phosphorylation ~1.5-fold and ~3.3-fold respectively, consistent with previous findings^6^ (Fig. 3, Supplementary Fig. 3). Treatment with the specific LRRK2 kinase inhibitor MLi-2 demonstrated LRRK2 kinase dependency of Rab10 phosphorylation as well as dephosphorylation of the Ser935 LRRK2 biomarker site, in keeping with response to Type 1 LRRK2 inhibitors such as MLi-2.

### Predicted impact of the *LRRK2* p.Arg1067Gln variant on the structure of LRRK2

The LRRK2 Arg1067 residue is highly conserved (Consurf score of 7/9^29^) and is located within the Leucine Rich Repeat (LRR) domain of LRRK2 upstream of the biomarker 14-3-3 binding phosphorylation sites.

**Fig. 4:**
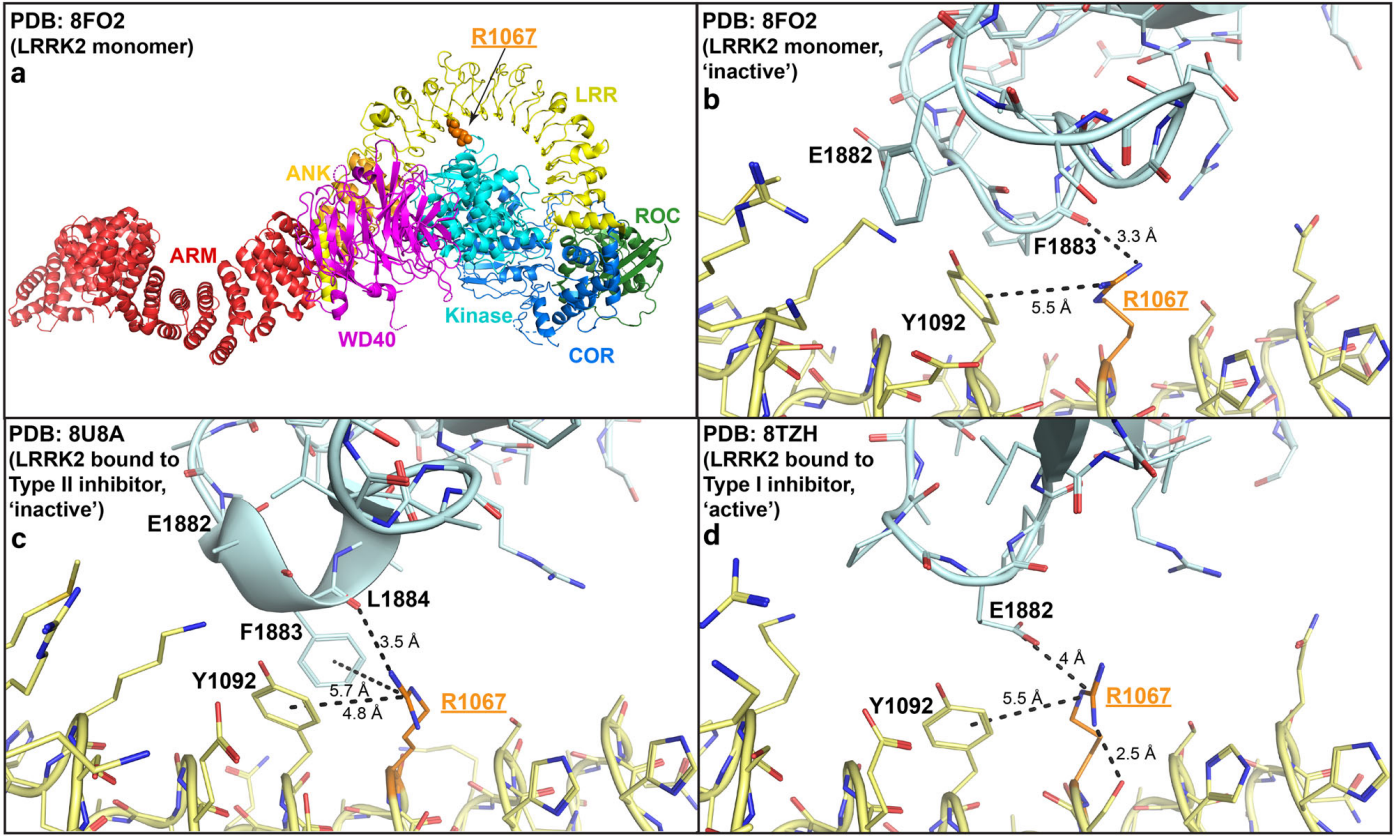
Structural insights into predicted impact of the *LRRK2* p.Arg1067Gln variant. Overview of the inactive LRRK2 monomer (**a**) with detailed view of the Arg1067Gln residue in the inactive LRRK2 monomer (PDB: 8FO2) (**b**), LRRK2 bound to Type II inhibitor that stabilizes LRRK2 kinase domain in the inactive conformation (PDB: 8U8A) (**c**), and LRRK2 bound to Type I inhibitor that stabilizes LRRK2 kinase domain in the active conformation (PDB: 8TZH) (**d**). The interactions that Arg1067 makes with kinase domain residues Phe1883 (electrostatic backbone and potentially Pi-stacking) and Leu1884 (electrostatic backbone) in the inactive conformation are highlighted (**b**, **c**). The interaction that Arg1067 makes with kinase domain residues Glu1882 (electrostatic) in the active conformation is also illustrated (**d**).

The LRR domain wraps over the LRRK2 kinase domain shielding it from interacting with substrates in the inactive conformation. Previous modeling suggested that the p.Arg1067Gln variant might destabilize the LRR-kinase domain interaction, leading to activation of LRRK2^6^. Analyzing the most recent cryogenic electron microscopy (Cryo-EM) studies of LRRK2 in the inactive and active conformations (Fig. 4), reveals that the p.Arg1067Gln mutation could impact both the inactive as well as active LRRK2 conformations. In the inactive conformations, the Arg1067 residue forms electrostatic backbone and potentially Pi-stacking interactions with kinase domain residue Phe1883 and electrostatic backbone interactions with Leu1884 (Fig. 4b, c). In the active LRRK2 structure, a conformational change induces a new electrostatic interaction of Arg1067 with kinase domain Glu1882 residue, that would also be impacted by the Arg1067Gln variant with subsequent functional impact on LRRK2 catalytic activity (Fig. 4d).

## Discussion

In the era of personalized precision medicine, determining the pathogenicity of single variants is crucial for properly interpreting and returning to patients and families the results of genetic testing, which is becoming increasingly commonplace^30–32^. Importantly also, genetics-informed therapies (e.g., LRRK2 kinase inhibitors) have entered phase 3 trials, and knowledge of the pathogenicity of different *LRRK2* variants is critical for patient selection and stratification^4^. Our data clearly show that the *LRRK2* p.Arg1067Gln variant activates LRRK2 kinase pathway activity, in vivo in peripheral blood monocytes from an affected variant carrier with PD compared to a control, as well as in a robust cellular overexpression system that allows evaluation of LRRK2 variant effect on LRRK2 kinase function. Furthermore, our data from in vitro overexpression in HEK293 cells show that the p.Arg1067Gln variant stimulates LRRK2 activity even more than the p.Gly2019Ser variant, suggesting that PD patients with this variant should be considered for ongoing LRRK2 clinical trials with kinase inhibitors. Based on the functional data and the enrichment in patients with an odds ratio for PD of 8.0, the ACMG PS3 criterion for “strong” evidence of pathogenicity of the p.Arg1067Gln variant is fulfilled^23^ and the *LRRK2* p.Arg1067Gln variant should be reclassified as pathogenic. Notably, this variant appears to be more common among East Asians, especially among patients of Chinese ancestry.

Interestingly, while the majority of pathogenic LRRK2 variants are located in the catalytic ROC-COR or kinase domains of the protein (and induce kinase hyperactivation)^33^, the p.Arg1067Gln variant is located within the LRR domain of LRRK2. Our modeling analysis indicates that the p.Arg1067Gln variant weakens the interaction between the LRR and the kinase domains in both the inactive and active LRRK2 conformations and we would predict that this enhances LRRK2 catalytic activity by facilitating access of Rab substrates to the LRRK2 kinase domain.

Regarding the clinico-demographic features of the p.Arg1067Gln variant carriers, several points are notable. Almost 90% were of East Asian ancestry (Chinese, Japanese, or Korean). It has been suggested that these three groups separated from each other from their recent common ancestor ~3000–3600 years ago^34^. The three “non-Asian” patients were two individuals from one family of Turkish origin, and one Italian. Although Turkey is geographically close to Europe, it is a “melting pot” of East and West, and it is believed that early Turks originated from Northeast Asia^35^. There was some evidence of a shared haplotype around the p.Arg1067Gln variant in two unrelated probands from mainland China, and further studies including more detailed haplotype analysis using whole genome sequencing (WGS) data of other p.Arg1067Gln carriers will shed light on the possibility of an ancient founder event(s), as has been shown for some of the more common *LRRK2* variants (p.Gly2019Ser and p.Arg1441Gly)^4,36,37^. Future work could also utilize increasingly available analytical tools that can provide automated and highly accurate ancestry predictions, rather than relying on patient self-report alone^38^. Although *LRRK2*-PD is often said to be clinically indistinguishable from “idiopathic” PD, the MDSGene database (https://www.mdsgene.org) interestingly reveals that a substantial proportion of patients with pathogenic or likely pathogenic *LRRK2* variants (265/863 = 31%) had early-onset PD (EOPD, defined as motor symptom onset below age 50 years). Our results are in line with this observation, with 9/22 (=41%) patients having EOPD.

The majority (14/18 = 78%) of patients were apparently sporadic, which is in keeping with the incomplete penetrance of pathogenic *LRRK2* variants (in our series, a variant-positive sibling was clinically unaffected at age 63 years, and an unaffected 83-year-old carrier has also been reported in the literature^16^). The p.Gly2019Ser variant, which has been the best studied *LRRK2* variant, was associated with a 49% cumulative incidence of PD by age 80 years in the largest study published to date^36^. The penetrance of pathogenic *LRRK2* variants highly depends on age, and is also influenced by ancestry/geography, genetic background such as modifier variants, as well as environmental factors^4,39–42^. Estimates may also differ on account of differences in healthcare access that can result in ascertainment bias^11,43^. Another possible reason for the low rate of familial history in our cases could be social stigma-related issues common in Asian societies resulting, for example, in a reluctance of probands and their families to disclose their PD diagnosis and to have other relatives brought in for assessment^30,44,45^. Further studies are needed to determine the penetrance of the p.Arg1067Gln variant.

Finally, it is notable that the first Malaysian patient enrolled in this series had a somewhat atypical presentation with impaired upgaze which initially raised concerns about possible progressive supranuclear palsy (PSP) (however, atypical features were not described in any of the other p.Arg1067Gln variant carriers). Pleiotropy with pathogenic *LRRK2* variants (e.g., p.Gly2019Ser, p.Arg1441Cys) has been recognized with, for example, isolated cases exhibiting clinical^9,46^ and/or pathological features of tauopathy/PSP^4,47,48^ and a substantial proportion lacking evidence of alpha-synucleinopathy^49,50^.

In summary, this report exemplifies how population-specific genetics in PD and functional evaluation at the variant level can help resolve the pathogenicity of *LRRK2* variants. For *LRRK2* p.Arg1067Gln, we recommend reclassification from VUS to “pathogenic” for PD. Moving forward, PD genetic testing strategies should include screening for this variant, especially in East Asians. Furthermore, since the p.Arg1067Gln variant appears to impact protein function more profoundly than, for example, p.Gly2019Ser, carriers of this variant should also be offered the opportunity to participate in clinical trials of new therapies targeting LRRK2 kinase hyperactivity. We anticipate that future systematic analyses of *LRRK2* variants via deep mutational scanning will likely shed light on many more variants of yet uncertain significance. Further, we highlight the value of globally diverse research to comprehensively understand the genetic architecture of PD^3,14,51^.

## Methods

### Literature review and database search

We conducted a PubMed search of PD cases with the p.Arg1067Gln variant published in English, using the search terms “Parkinson’s disease” in combination with “LRRK2 R1067Q”, and a search of the MDSGene PARK-*LRRK2* database (https://www.mdsgene.org/genes/PARK-LRRK2) for “c.3200G>A”. We also checked the reference lists in relevant articles.

We further queried our Malaysian, Singaporean, and mainland Chinese PD databases for the p.Arg1067Gln variant (total number of samples 4901, of which 4081 [83%] were from patients of Chinese ancestry). Malaysian samples (*n* = 1871, of whom 1251 were Chinese) were tested via a variety of genetic testing platforms, including commercial (CENTOGENE) PD gene panel, genotyping via NBA and/or WGS (the latter two via the Global Parkinson’s Genetics Program, GP2)^24,26,52^. Next-generation sequencing was employed for the samples from Singapore (whole exome sequencing [WES]^53^ for *n* = 2430, of whom 2230 were Chinese) and mainland China (*n* = 600; 53% WES and 44% WGS; the remainder undergoing targeted sequencing).

Forty-one SNPs over a 140kbp interval across the p.Arg1067Gln variant were extracted from the seven Malaysian and Singaporean NBA/CENTOGENE/whole exome datasets, 307 Malaysian EAS controls, and 1220 Malaysian EAS PD patients without p.Arg1067Gln, and haplotypes inferred using Beagle 5.4 using default settings^54^. Additionally, SNPs across a 2 Mb region were extracted from whole genome sequencing datasets from proband III:1 from Family 1 and proband II:1 from Family 3, and from 10 PD patients negative for the p.Arg1067Gln variant, followed by inspection of genotypes across the interval to infer shared haplotypes.

### Ethical approvals and subject recruitment

All studies involving human subjects were reviewed and approved by the respective Institutional Ethics Committees. The recruitment of Malaysian samples was approved by the Medical Research Ethics Committee (MREC) of the University of Malaya Medical Centre (UMMC; MREC ID no. 2022427-11195), while the Singaporean study received approval from the SingHealth Centralised Institutional Review Board (CIRB Ref: 2019/2013 and Ref: 2019/2330). Mainland Chinese samples were granted approval by the Second Affiliated Hospital, Zhejiang University School of Medicine (Approval ID: A2023734). All studies adhered to the Declaration of Helsinki, and written informed consent was obtained from all participants.

### Monocyte isolation from fresh peripheral blood

Peripheral blood mononuclear cells express relatively high levels of LRRK2 as well as Rab10 and are therefore suited for interrogating the LRRK2 kinase pathway in human participants. 20 mL of blood was collected from the Malaysian *LRRK2* p.Arg1067Gln variant carrier with PD (PD-3402) and an age-matched healthy control for immediate isolation of peripheral blood monocytes via immunomagnetic negative selection as described before^27,28^. Cells were then lysed, snap frozen and shipped for further processing for multiplexed quantitative immunoblotting for LRRK2 kinase pathway activation and analysis thereof at the Medical Research Council Protein Phosphorylation and Ubiquitylation Unit, University of Dundee, Dundee, United Kingdom.

### Multiplexed quantitative immunoblotting for LRRK2 kinase pathway activation

Cell lysates were prepared at a concentration of 2 µg/µL in NuPage LDS Sample Buffer (×4) with 5% β-mercaptoethanol and boiled at 96 °C for 10 min for SDS-PAGE, and LICOR quantitative immunoblotting as described before^6^. The following primary antibodies were used: multiplexed anti-LRRK2 mouse (NeuroMab #75-253) and anti-pS935 rabbit (Abcam #ab133450) monoclonal antibodies at a 1:1000 dilution (1 µg/mL), GAPDH mouse monoclonal antibody (Santa Cruz Biotechnology #sc-32233) diluted 1:2000 (50 ng/mL) and multiplexed anti-Rab10 mouse (Nanotools #0680-100/Rab10) and anti-MJFF-pRab10 rabbit (Abcam #ab230261) monoclonal antibodies at a 1:1000 dilution (1 µg/mL). The following multiplexed fluorescent secondary antibodies were used: multiplexed 1:10,000 goat anti-mouse IRDye 680LT and 1:10,000 goat anti-rabbit IRDye 800CW antibodies. All blots and gels were derived from the same experiment and were processed in parallel.

### HEK293 transient overexpression system and plasmids

Detailed protocols that describe transfection and lysis of HEK293 cells were used as before^6^. The following plasmids (all pCMV5) used in this study were obtained from the MRC PPU Reagents and Services (https://mrcppureagents.dundee.ac.uk): Flag-empty (DU 44060), Flag LRRK2 wildtype (DU6841), Flag LRRK2 D2017A (kinase inactive), Flag LRRK2 R1067Q (DU13043), and Flag LRRK2 R1441G (DU13077). Three independent biological replicates, including one replicate where cells were treated with 200 nM MLi-2 or 0.1% (v/v) DMSO (vehicle), were treated for 1.5 h before cell lysis.

### Modeling of the impact of the p.Arg1067Gln variant on LRRK2 structure

The most recent Cryo-EM structures of LRRK2^6^ were obtained from PDB (8FO2, 8TZH, 8U8A) and visualized using PyMOL 3.

## Supplementary information

Supplementary Information - Clinical and functional evidence for the pathogenicity of the LRRK2 p.Arg1067Gln variant

Supplementary Table 4

## Data Availability

The datasets generated and analyzed during the current study are available from the corresponding authors upon request.
