# Supplementary material for: Clinical and functional evidence for the pathogenicity of the LRRK2 p.Arg1067Gln variant
Source: NPJ Parkinsons Dis. 2025 Feb 23;11:34. doi: 10.1038/s41531-025-00884-6 (PMC11847920; doi:10.1038/s41531-025-00884-6)
Supplement: Supplementary file 1 — Supplementary Information - Clinical and functional evidence for the pathogenicity of the LRRK2 p.Arg1067Gln variant [file 41531_2025_884_MOESM1_ESM.docx]

**Clinical and functional evidence for the pathogenicity of the *LRRK2* p.Arg1067Gln variant**

**
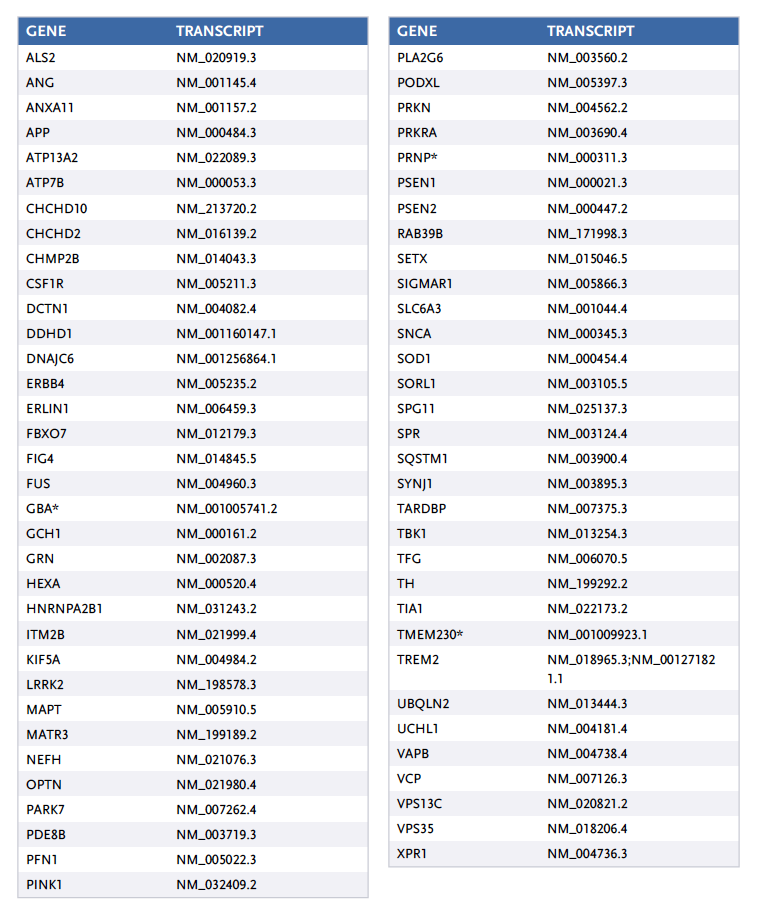
**

**Supplementary Figure 1. Invitae Hereditary Parkinson Disease and Parkinsonism Panel (as of 17 November 2022)**


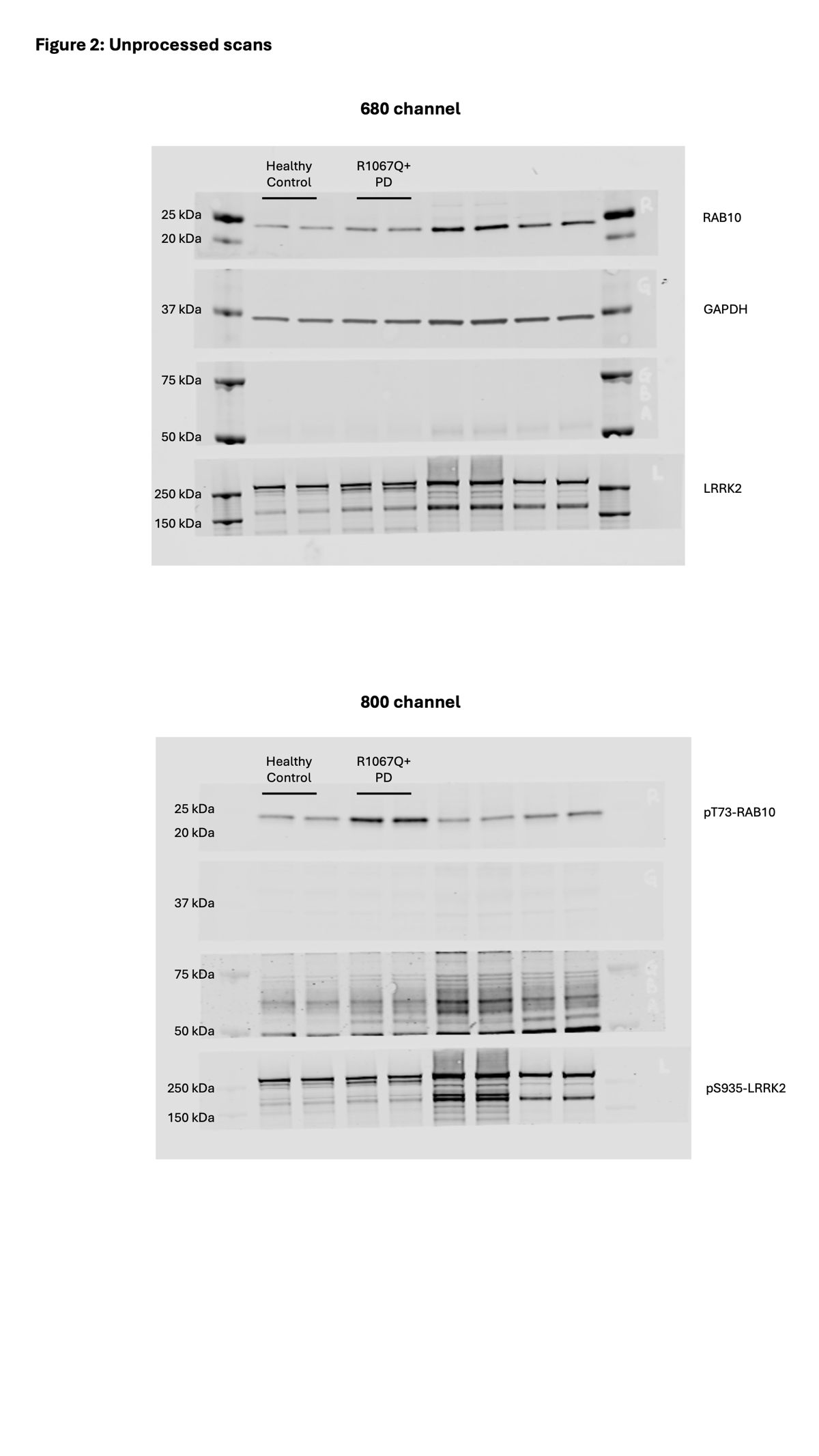


**Supplementary Figure 2: Unprocessed western blot scans corresponding to Figure 2.**

**
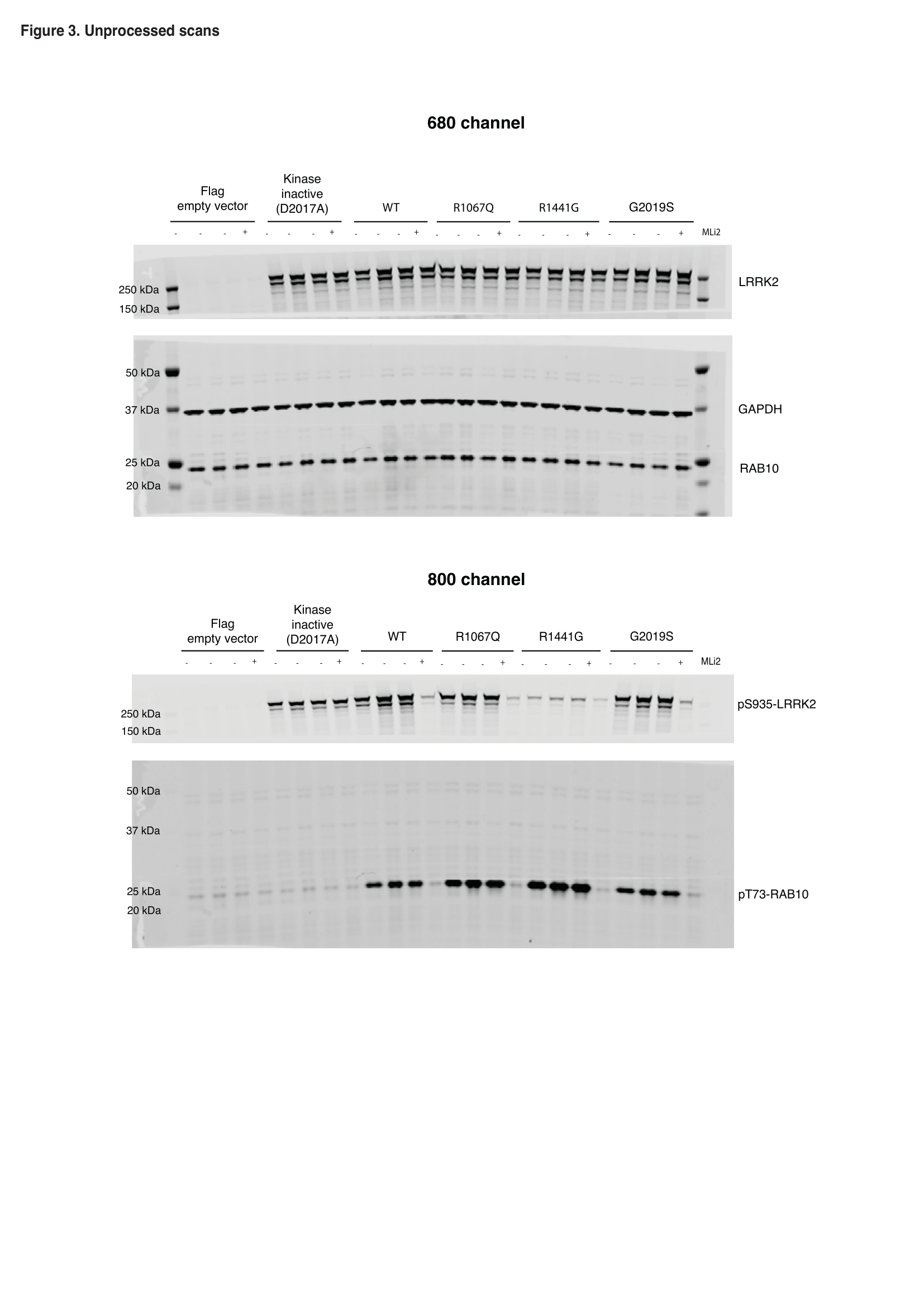
**

**Supplementary Figure 3: Unprocessed western blot scans corresponding to Figure 3.**

**Supplementary Table 1. Motor and non-motor features of patients harbouring the *LRRK2* p.Arg1067Gln variant.**

| Paper | Skipper et al. *Neurol.* 2005 | Zabetian et al. *Mov Disord*. 2009 | Kessler et al. *Parkinsonism Rel Disord*. 2017 | Youn et al. *Neurobiol Aging*. 2019 | Yang et al. *Neurobiol Aging*. 2019 | Li et al. *Neurobiol Aging*. 2020 | Zheng et al. *Mol Genet Genomic Med*. 2020 | Salemi et al. *biomedicines.* 2023. | This report:  Patients 1-3 (Malaysian) (PD-3402, PD-1785, & PD-330, respectively) | This report:  Patients 4-7 (Singaporean) (P1785, P199, P694, & PD/LT 16, respectively) | This report:  Patients 8-12 (mainland Chinese) | Overall, *n*=23 cases |
| --- | --- | --- | --- | --- | --- | --- | --- | --- | --- | --- | --- | --- |
| Bradykinesia | ✓ | UNK  UNK | ✓  ✓ | ✓ | ✓  ✓ | UNK | ✓ | ✓ | ✓  ✓  ✓ | 🗶  UNK  ✓  ✓ | ✓  UNK  ✓  ✓  ✓ | 17✓,1🗶,5UNK |
| Rigidity | ✓ | UNK  UNK | ✓  ✓ | UNK | ✓  ✓ | UNK | UNK | UNK | ✓  UNK  ✓ | 🗶  UNK  ✓  ✓ | ✓  UNK  ✓  ✓  ✓ | 13✓,1🗶,9UNK |
| Tremor | ✓ | UNK  UNK | ✓  ✓ | UNK | ✓  🗶 | ✓ | UNK | ✓ | ✓  ✓  ✓ | 🗶  UNK  🗶  ✓ | 🗶  UNK  ✓  ✓  🗶 | 12✓,5🗶,6UNK |
| Gait difficulty | UNK | UNK  UNK | UNK  UNK | UNK | UNK  UNK | UNK | UNK | UNK | ✓  ✓  ✓ | 🗶  UNK  ✓  UNK | ✓  UNK  UNK  ✓  ✓ | 7✓,1🗶,15UNK |
| Gait freezing | UNK | UNK  UNK | UNK  UNK | UNK | UNK  UNK | UNK | UNK | UNK | ✓  ✓  UNK | 🗶  UNK  ✓  ✓ | ✓  UNK  🗶  UNK  🗶 | 5✓,3🗶,15UNK |
| PI / Falls | UNK | UNK  UNK | UNK  UNK | ✓ | ✓  ✓ | 🗶 | UNK | UNK | ✓  ✓  🗶 | 🗶  UNK  UNK  UNK | ✓  UNK  🗶  UNK  🗶 | 6✓,5🗶,12UNK |
| Dystonia | 🗶 | UNK  UNK | UNK  UNK | 🗶 | UNK  UNK | 🗶 | UNK | UNK | 🗶  ✓ (facial)  UNK | 🗶  UNK  UNK  UNK | 🗶  UNK  🗶  🗶  🗶 | 1✓,9🗶,13UNK |
| Obvious asymmetry | ✓ | UNK  UNK | ✓  ✓ | UNK | UNK  UNK | UNK | UNK | UNK | ✓  🗶  🗶 | UNK  UNK  UNK  UNK | ✓  UNK  ✓  UNK  ✓ | 7✓,2🗶,14UNK |
| Diurnal variation | UNK | UNK  UNK | UNK  UNK | UNK | UNK  UNK | UNK | UNK | UNK | 🗶  UNK  UNK | 🗶  UNK  UNK  UNK | UNK  UNK  UNK  UNK  UNK | 0✓,2🗶,21UNK |
| Sleep benefit | UNK | UNK  UNK | UNK  UNK | UNK | UNK  UNK | UNK | UNK | UNK | 🗶  UNK  UNK | 🗶  UNK  UNK  UNK | UNK  UNK  UNK  UNK  UNK | 0✓,2🗶,21UNK |
| UMN signs | UNK | UNK  UNK | UNK  UNK | UNK | UNK  UNK | UNK | UNK | UNK | 🗶  UNK  🗶 | 🗶  UNK  🗶  UNK | UNK  UNK  UNK  UNK  🗶 | 0✓,5🗶,18UNK |
| Clear response to DRT | ✓ | UNK  UNK | ✓ (patient was also treated with DBS @45y)  ✓ | N/A (drug-naïve) | ✓  ✓ | ✓ | UNK | N/A (drug-naïve) | ✓  ✓  ✓ | ✓  ✓  ✓  UNK | ✓  UNK  ✓  N/A (drug-naïve)  ✓ | 15✓,0🗶,5UNK,3N/A |
| MF | ✓ | UNK  UNK | UNK  UNK | N/A | UNK  UNK | ✓ | UNK | N/A (drug-naïve) | ✓ (S)  ✓ (S) - offered DBS  ✓ | 🗶  UNK  ✓  UNK | ✓  UNK  ✓  N/A (drug-naïve)  ✓ | 9✓,1🗶,10UNK,3N/A |
| LID | ✓ | UNK  UNK | UNK  UNK | N/A | ✓  🗶 | 🗶 | UNK | N/A (drug-naïve) | ✓  ✓  🗶 | UNK  UNK  UNK  UNK | ✓  UNK  UNK  N/A (drug-naïve)  🗶 | 5✓,4🗶,11UNK,3N/A |
| RBD | 🗶 | UNK  UNK | UNK  UNK | 🗶 | UNK  UNK | UNK | UNK | UNK | ✓  UNK  🗶 | UNK  UNK  UNK  UNK | ✓  UNK  ✓  UNK  🗶 | 3✓,4🗶,16UNK |
| Insomnia | 🗶 | UNK  UNK | UNK  UNK | UNK | UNK  UNK | UNK | UNK | UNK | ✓  ✓  🗶 | 🗶  UNK  UNK  UNK | ✓  UNK  UNK  UNK  ✓ | 4✓,3🗶,16UNK |
| EDS | UNK | UNK  UNK | UNK  UNK | UNK | UNK  UNK | UNK | UNK | UNK | 🗶  UNK  🗶 | UNK  UNK  UNK  UNK | UNK  UNK  UNK  UNK  UNK | 0✓,2🗶,21UNK |
| Depression | 🗶 | UNK  UNK | UNK  UNK | ✓ (BDI=16) | UNK  UNK | 🗶 | UNK | UNK | ✓  UNK  🗶 | UNK  UNK  UNK  UNK | ✓  UNK  UNK  UNK  UNK | 3✓,3🗶,17UNK |
| Anxiety | 🗶 | UNK  UNK | UNK  UNK | UNK | UNK  UNK | UNK | UNK | UNK | ✓  ✓  🗶 | ✓  UNK  🗶  UNK | ✓  UNK  UNK  UNK  UNK | 4✓,3🗶,16UNK |
| MCI | 🗶 | UNK  UNK | UNK  UNK | ✓ (MoCA=19) | UNK  UNK | 🗶 (MMSE=30) | UNK | ✓ | 🗶  🗶  ✓ | UNK  UNK  UNK  UNK | ✓  UNK  UNK  UNK  🗶 | 4✓,5🗶,14UNK |
| Dementia | 🗶 | UNK  UNK | 🗶  🗶 | UNK | 🗶  🗶 | 🗶 | UNK | 🗶 | 🗶  🗶  🗶 | UNK  UNK  UNK  ✓ | 🗶  UNK  UNK  UNK  🗶 | 1✓,12🗶,10UNK |
| VH | UNK | UNK  UNK | UNK  UNK | UNK | UNK  UNK | UNK | UNK | UNK | ✓  UNK  🗶 | UNK  UNK  UNK  UNK | 🗶  UNK  UNK  UNK  UNK | 1✓,2🗶,20UNK |
| ICBs | UNK | UNK  UNK | UNK  UNK | UNK | UNK  UNK | UNK | UNK | UNK | 🗶  UNK  🗶 | UNK  UNK  UNK  UNK | 🗶  UNK  UNK  UNK  UNK | 0✓,3🗶,20UNK |
| Constipation | UNK | UNK  UNK | UNK  UNK | ✓ | UNK  UNK | UNK | UNK | UNK | ✓  ✓  ✓ | UNK  UNK  UNK  UNK | ✓  UNK  ✓  ✓  ✓ | 8✓,0🗶,15UNK |
| Urinary dysfunction | UNK | UNK  UNK | UNK  UNK | UNK | UNK  UNK | UNK | UNK | UNK | ✓  UNK  ✓ | UNK  UNK  UNK  UNK | ✓  UNK  UNK  UNK  UNK | 3✓,0🗶,20UNK |
| OH | 🗶 | UNK  UNK | UNK  UNK | UNK | UNK  UNK | UNK | UNK | UNK | 🗶  UNK  🗶 | UNK  UNK  UNK  ✓ | UNK  UNK  UNK  UNK  UNK | 1✓,3🗶,19UNK |
| Pain | UNK | UNK  UNK | UNK  UNK | UNK | UNK  UNK | UNK | UNK | UNK | ✓  UNK  🗶 | UNK  UNK  UNK  UNK | 🗶  UNK  UNK  UNK  UNK | 1✓,2🗶,20UNK |
| Hyposmia | UNK | UNK  UNK | UNK  UNK | ✓ | UNK  UNK | UNK | UNK | UNK | 🗶  UNK  UNK | UNK  UNK  UNK  UNK | 🗶  UNK  ✓  🗶  ✓ | 3✓,3🗶,17UNK |
| Underweight | UNK | UNK  UNK | UNK  UNK | UNK | UNK  UNK | UNK | UNK | UNK | 🗶  UNK  UNK | UNK  UNK  UNK  UNK | 🗶  UNK  UNK  UNK  UNK | 0✓,2🗶,21UNK |
| Atypical features | 🗶 | UNK  UNK | 🗶  🗶 | UNK | UNK  UNK | UNK | UNK | UNK | ✓  🗶  🗶 | 🗶  🗶  🗶  UNK | 🗶  UNK  🗶  UNK  🗶 | 1✓,11🗶11UNK |

**Abbreviations.** PI=postural instability; UMN=upper motor neuron; DRT=dopamine replacement therapy; MF=motor fluctuations; LID=levodopa-induced dyskinesias; RBD=REM sleep behaviour disorder; EDS=excessive daytime sleepiness; MCI=mild cognitive impairment; VH=visual hallucinations; ICB=impulsive-compulsive behaviours; OH=orthostatic hypotension; UNK=unknown.

**Supplementary Table 2. Comparison of mutant allele frequencies (c.3200G>A) between Chinese-ancestry patients with Parkinson’s disease and East Asian controls.**

|  | **Chinese PD**  (n=4,081) | **Controls**  (n=22,734)  (gnomAD EAS Controls n=22,427 + Malaysian Chinese Controls n=307) |
| --- | --- | --- |
| Wildtype allele (G) | 8,152 | 45,461 |
| Mutant allele (A) | 10 | 7 |
| Total | 8,162 | 45,468 |
| Minor allele frequency | 0.00123 | 0.00015 |
| Odds ratio (95% CI)  P value | 7.97 (3.03 - 20.94)  P<0.0001 | |

**Supplementary Table 3. Haplotype analysis of a 140kbp region encompassing the *LRRK2* p.Arg1067Gln variant (in bold), located on chromosome 12 in the Malaysian and Singaporean carriers, inferred using Beagle 5.4.** Chromosomal (NC_000012.12) and genomic (NG_011709.1) locations are listed for each SNP. Minor allele frequencies (MAF) in the East Asian (EAS) population were obtained from gnomAD v.4.1. Alternate alleles are highlighted in grey.

| **Chromosomal location (Chr12: NC_000012.12)** | **Genomic location (NG_011709.1), wildtype>alternate allele** | **SNP ID** | **MAF EAS** | **Patient 1 (PD-3402)** | **Patient 2 (PD-1785)** | **Patient 3 (PD-330)** | **Patient 4 (P1785)** | **Patient 5 (P199)** | **Patient 6 (P694)** | **Patient 7 (PD/LT 16)** |
| --- | --- | --- | --- | --- | --- | --- | --- | --- | --- | --- |
| 40222612 | g.2602A>G | rs28365207 | 0.1233 | A | A | A | A | A | A | A |
| 40227006 | g.6996C>G | rs1491942 | 0.3583 | G | C | C | C | C | C | C |
| 40237989 | g.17979T>C | rs10878245 | 0.3513 | T | T | T | T | T | T | T |
| 40258718 | g.38708T>C | rs2723264 | 0.8366 | C | C | C | C | C | C | C |
| 40262377 | g.42367A>G | rs11175769 | 0.3515 | A | A | A | A | A | A | A |
| 40263898 | g.43888C>G | rs7308720 | 0.1218 | C | C | C | C | C | C | C |
| 40276592 | g.56582G>A | rs7971935 | 0.02765 | G | G | G | G | G | G | G |
| 40278187 | g.58177A>G | rs10878307 | 0.02105 | A | A | A | A | A | A | A |
| 40283897 | g.63887C>T | rs34410987 | 0.006246 | C | C | C | C | C | C | C |
| 40283947 | g.63937C>T | rs376015112 | 0.00002229 | C | C | C | C | C | C | C |
| 40284011 | g.64001G>A | rs35173587 | <0.01 | G | G | G | G | G | G | G |
| 40285609 | g.65599G>A | rs117165831 | <0.01 | G | G | G | G | G | G | G |
| 40293624 | g.73614G>A | rs58559150 | <0.01 | G | G | G | G | G | G | G |
| 40293896 | g.73886C>T | rs5006481 | 0.2716 | C | C | C | C | C | C | C |
| 40294866 | g.74856G>T | rs17519916 | <0.01 | G | G | G | G | G | G | G |
| 40294893 | g.74883T>C | rs7966550 | 0.1561 | T | T | T | T | T | T | T |
| 40295466 | g.75456G>A | rs75148313 | <0.01 | G | G | G | G | G | G | G |
| **40298346** | **g.78336G>A** | **rs111341148** | **0.0001561** | **A** | **A** | **A** | **A** | **A** | **A** | **A** |
| 40298479 | g.78469G>T | rs78365431 | 0.0001338 | G | G | G | G | G | G | G |
| 40303632 | g.83622G>A | rs10784498 | 0.1889 | G | G | G | G | G | G | G |
| 40304141 | g.84131C>T | rs41286480 | 0.00004462 | C | C | C | C | C | C | C |
| 40305791 | g.85781C>G | rs4640000 | <0.01 | C | C | C | C | C | C | C |
| 40305946 | g.85936T>A | rs183902574 | <0.01 | T | T | T | T | T | T | T |
| 40308481 | g.88471G>A | rs72546338 | 0.0001561 | G | G | G | G | G | G | G |
| 40308618 | g.88608A>G | rs17466213 | 0.0001338 | A | A | A | A | A | A | A |
| 40309109 | g.89099G>A | rs7133914 | 0.1178 | G | G | G | G | G | G | G |
| 40309145 | g.89135C>T | rs72546327 | 0.00004458 | C | C | C | C | C | C | C |
| 40309174 | g.89164G>C | rs113589830 | <0.01 | G | G | G | G | G | G | G |
| 40309185 | g.89175G>A | rs11175964 | 0.1177 | G | G | G | G | G | G | G |
| 40310450 | g.90440C>T | rs74681492 | 0.004702 | C | C | C | C | C | C | C |
| 40313976 | g.93966G>A | rs35507033 | <0.01 | G | G | G | G | G | G | G |
| 40314059 | g.94049C>T | rs33958906 | 0.0001562 | C | C | C | C | C | C | C |
| 40320032 | g.100022C>A | rs1427263 | 0.5084 | C | C | C | C | C | C | C |
| 40320043 | g.100033G>C | rs33949390 | 0.01022 | G | C | G | C | G | G | G |
| 40320071 | g.100061A>G | rs11176013 | 0.479 | A | A | A | A | A | A | A |
| 40320099 | g.100089T>A | rs11564148 | 0.3232 | T | T | T | T | T | T | T |
| 40348452 | g.128442G>A | rs10878405 | 0.4791 | G | G | G | G | G | G | G |
| 40350973 | g.130963A>C | rs7303525 | 0.1883 | A | A | A | A | A | A | A |
| 40351006 | g.130996G>A | rs7132187 | 0.2051 | G | G | G | G | G | G | G |
| 40363526 | g.143516G>A | rs34778348 | 0.02628 | G | G | G | G | G | G | G |
| 40363528 | g.143518A>G | rs33962975 | 0.01639 | A | A | A | A | A | A | A |

**Supplementary Table 5. Genotypes from a 110463bp region encompassing the *LRRK2* p.Arg1067Gln variant (rs111341148 in bold), in Proband III:1 (Family 1) and proband II:1 (Family 3), and ten PD patients without the p.Arg1067Gln variant.**

Chromosomal (NC_000012.12) locations are listed for each SNP. Minor allele frequencies (MAF) in the East Asian (EAS) population were obtained from gnomAD v.4.1. The ten patients without the p.Arg1067Gln variant are coded as PDc1-10. Heterozygous and homozygous alternate genotypes are highlighted in grey. The shared haplotype between Proband III:1 and Proband II:1 is shaded in blue.

| **Chromosomal location (Chr12: NC_000012.12)**  **wildtype>**  **alternate allele** | **SNP ID** | **MAF EAS** | **Proband III:1 (Family 1)** | **Proband II:1**  **(Family 3)** | PD  c1 | PDc2 | PDc3 | PDc4 | PDc5 | PDc6 | PDc7 | PDc8 | PDc9 | PDc10 |
| --- | --- | --- | --- | --- | --- | --- | --- | --- | --- | --- | --- | --- | --- | --- |
| 40237989; T>C | rs10878245 | 0.3513 | TC | TC | TC | CC | TC | TC | TC | TC | TT | TT | TT | CC |
| 40258718; C>T | rs2723264 | 0.1634 | CC | CC | CC | TT | TC | TT | CC | CC | TC | TT | TC | CC |
| 40262377, A>G | rs11175769 | 0.3515 | AG | AG | AG | AA | AG | AG | AG | AG | AA | AA | AA | AA |
| 40276902; G>A | [rs139952019](http://www.ncbi.nlm.nih.gov/snp/rs139952019) | 0.09675 | GA | GA | GG | GG | GG | GG | GG | GG | GG | GG | GG | GG |
| 40291358; A>G | [rs7953822](http://www.ncbi.nlm.nih.gov/snp/rs7953822) | 0.0000 | AA | AA | AA | GG | GG | GG | GG | GG | GG | GG | GG | GG |
| **40298346; G>A** | **rs111341148** | **0.0001561** | **GA** | **GA** | GG | GG | GG | GG | GG | GG | GG | GG | GG | GG |
| 40313477; G>A | [rs11564178](http://www.ncbi.nlm.nih.gov/snp/rs11564178) | 0.09722 | GA | GA | GG | GG | GG | GG | GG | GG | GG | GG | GG | GG |
| 40320032; C>A | rs1427263 | 0.5084 | CA | CA | CA | AA | CA | AA | CA | AA | CA | CC | CA | AA |
| 40320071; A>G | rs11176013 | 0.479 | AG | AG | AG | GG | AG | GG | AG | AG | AA | AA | AG | GG |
| 40320099; T>A | rs11564148 | 0.3232 | TA | TA | TA | AA | TA | TA | TA | TA | TT | TT | TT | AA |
| 40348452; G>A | rs10878405 | 0.4791 | GA | GA | GA | AA | GA | GA | GA | GG | GG | GG | GG | AA |
